# Retraction: Prevalence, predictors, and clinical presentation of acute pericardial effusion following percutaneous coronary intervention

**DOI:** 10.3389/fcvm.2024.1425882

**Published:** 2024-05-08

**Authors:** Frontiers Editorial Office

A Retraction of the Original Research Article Prevalence, predictors, and clinical presentation of acute pericardial effusion following percutaneous coronary intervention by Zhao B, Zhang J, Li Y, Feng X, Mao S, Yin Z, Liu L, Song D and Wang S (2022). Front. Cardiovasc. Med. 8:759164. doi: 10.3389/fcvm.2021.759164

Following publication, concerns were raised regarding the ethical consent and approval of this study. An investigation was conducted in accordance with Frontiers’ policies.

It was confirmed that the study lacked appropriate ethical approval and consent to publish identifiable data. This is a breach of Frontiers’ and the Committee on Publication Ethics’ guidelines; as such, this article is retracted. To protect the participants, any potentially identifiable data have been removed from the article.

This retraction was approved by the Chief Editor of Frontiers in Cardiovascular Medicine and the Chief Executive Editor of Frontiers. The authors did not agree to this retraction.

